# The MYTHS of *De novo* Crohn’s Disease After Restorative Proctocolectomy with Ileal Pouch-anal Anastomosis for Ulcerative Colitis

**Published:** 2020-03-11

**Authors:** SD James, AT Hawkins, JW Um, BR Ballard, DT Smoot, AE M’Koma

**Affiliations:** 1 Department of Pathology, Meharry Medical College School of Medicine, Nashville General Hospital, Nashville, Tennessee, United States; 2 Department of Pathology, Microbiology, and Immunology, Tennessee Valley Health Systems VA, Medical Center, Vanderbilt University Medical Center, Nashville, Tennessee, United States; 3 Division of General Surgery, Section of Colon and Rectal Surgery, Vanderbilt University School of Medicine, Nashville, Tennessee, United States; 4 Department of Surgery, Korea University College of Medicine, Seoul, South Korea; 5 Department of Pathology, Meharry Medical College School of Medicine, Nashville General Hospital, Nashville, Tennessee, United States; 6 Department of Medicine, Meharry Medical College School of Medicine, Nashville General Hospital, Nashville, Tennessee, United States; 7 Department of Surgery and Surgical Sciences, Meharry Medical College School of Medicine, Nashville, Tennessee, United States; 8 The American Society of Colon and Rectal Surgeons (ASCRS), Arlington Heights, IL 60005, United States; 9 The American Gastroenterological Association (AGA), Bethesda, MD 20814, United States

**Keywords:** Ulcerative colitis, Proctocolectomy, Ileal pouch-anal anastomosis, *De novo* Crohn’s Disease, Transformation, Conversion, Change of diagnosis, Human alpha-defensin 5

## Abstract

**Background:**

Inflammatory Bowel Disease (IBD) are the manifestation of overzealous dys-regulated immune response in the intestinal tract, directed primarily against the indigenous microbes combined with defective functioning of anti-inflammatory pathways. Finding a trustable lead to predicting *de novo* Crohn’s Disease (CD) prior to performing “pouch surgery”, Restorative Proctocolectomy (RPC) with Ileal Pouch-Anal Anastomosis (IPAA) for UC and/or Indeterminate Colitis (IC) is clinically important and remains debatable. *De novo* CD is a subsequent long-term postoperative complication in IBD patients with Ulcerative Colitis (UC) undergoing IPAA. Herewith we discuss this understanding in laboratory-based basic science research, with its molecular application as a possible corner stone tool for clinical progress and success in the IBD Clinic. Crypt Paneth cell (PCs) secreted enteroendocrine alpha-defensin 5 (*DEFA5*)” if developed properly is likely to solve diagnostic and prognostic difficulty in IBD Clinics. *DEFA5* has shown the ability to differentiate the predominant subtypes of colonic IBD (CC *vs*. UC) at first endoscopy biopsy, avoiding diagnosis delay prior to colectomy. In addition, *DEFA5* accurately circumvents indeterminate colitis (IC) patients into accurate IBD subtype (UC or CC). Further, *DEFA5* can be used in selecting CC patients that may have positive outcomes after IPAA surgery [1]. Furthermore, likewise, *DEFA5* can predict UC patients likely to have positive or poor outcome, e.g. those patients that are likely to transform/ convert and adhere to *de novo* Crohn’s after IPAA can be picked up in endoscopy biopsy before surgery.

**Aim:**

To assessed comprehensive state-of-the-art understanding domains on the *de novo* Crohn’s disease subsequent to IPAA surgery for ulcerative colitis.

**Methods:**

A literature search based on preferred reporting items for over-review and meta-analysis protocols (PRISMA-P) was performed. A comprehensive current search of PubMed, MEDLINE, CINAHL, Embase, Google^®^ search engine and Cochrane Database of collected reviews was performed from January 1990 through December 2018. The search consists of retrospective studies and case reports of reporting postoperative *de novo* CD incidence and adverse events. Secondary and hand/manual searches of reference lists, other studies cross-indexed by authors, reviews, commentaries, books and meeting abstracts were also performed. Studies were included only if the diagnosis of *de novo* CD was established clinically and histologically based on inflammation of afferent limb(s) or perianal disease. The search excluded non-English language and non-human studies as well as editorials.

**Results:**

Published data on *de novo* CD developing after RPC with IPAA are still limited. A total of three hundred and sixty-five (#365) patients in 13 publications reported *de novo* CD after a median follow-up of 66 (range: 3–236) months. All patients were diagnosed with clinically active pouch CD during follow-up surveillance after IPAA for UC or IC. A *de novo* CD diagnosis depended on either inflammation in the mucosa involving the small intestine proximal to the ileal pouch any time after IPAA surgery and/or when perianal complications developed after closure of a temporary diverting loop ileostomy. Successful management is facilitated by co-operation within a multidisciplinary team of gastroenterologists and colorectal surgeons and closely involving the patient in therapeutic decisions. Awareness of symptoms leads to timely consultation, diagnosis, treatment and restoration of intestinal continuity.

**Conclusion:**

The nature history and risk of *de novo* CD after IPAA for UC remains debatable. Chronic pouchitis and/or pouch failure often precedes a diagnosis of *de novo* CD. A successful management is facilitated by a triad cooperation between gastroenterologists, colorectal surgeons and the patient.

## 5. Introduction

Restorative Proctocolectomy (RPC) with Ileal Pouch-Anal Anastomosis (IPAA) is indicated in approximately 20% to 30% as the current recommended surgical standard procedure for curative treatment for suitable patients with medically refractory UC and IC predicted as UC [1–3]. Ulcerative colitis and IC designated UC generally, demonstrate acceptable functional results after RPC with IPAA [4, 5], but approximately 5 to 10 per cent of IPAA patients are subsequently diagnosed with *de novo* CD, leading to increased morbidity and rates of pouch failure [1, 5–9]. In preparation, prior to RPC with IPAA surgery patients must be well-informed, counseled and guided about *de novo* CD, given the fact that many patients view it as a curative surgery [10]. *De novo* CD is one of the most devastating complications of IPAA, which ultimately increases the risk for pouch malfunctions [11–15]. Restorative proctocolectomy with IPAA is usually contraindicated in patients with authentic colonic CD, also known as Crohn’s Colitis (CC), because of high risks and vulnerability for disease recurrence, fistulas, abscesses, and strictures which may lead to a significant higher incidence of pouch failure and pouch excision [16–18]. However, in highly selected patients with CC, RPC with IPAA has been indicated with positive outcomes [7, 19, 20]. Therefore, validation of the diagnostic potential using *DEFA5*, a newly found reliable marker to identify CC patients (Crohn’s-like clinically but have lower *DEFA5* levels) as well as IC patients who are potential candidates for this sphincter-preserving operation, is recommended. While RPC with IPAA is acceptable for patients with UC [2] or IC predicted as UC [21, 22], it should not be the first option for treating patients with CC. Identifying robust biomarkers predicting *de novo* CD among patients with UC and IC is clinically relevant and would improve surgical decision making. This underscores the critical need for predictive and precision medicine that can optimize diagnosis and disease management, provide more cost-effective strategies, and minimize the risk of adverse practices. Our experience in patients that were identified with pathologic features associated with UC-like subgroup of CC [PMID: 25278708] and those whose diagnoses clinically changed from UC to *de novo* CD [1] after IPAA surgery, all showed prominent *DEFA5* staining compared to those whose diagnosis remained unchanged. This is an indication that *DEFA5*, if well developed, could be used as a tool to predict UC patients likely to transform and convert and adhere to *de novo* CD prior to IPAA surgery. In this review of a field of vision narrative, we summarize the current status of prediction of *de novo* CD risk, clinical course, investigations, and response to treatment based on clinical case presentations. We also discuss concisely the potential and limitations of the currently used strategies.

## 6. Materials and Methods

A literature review was performed according to the preferred reporting items for over-review and meta-analysis protocols (PRISMA-P) guidelines [23]. A search of comprehensive reporting of UC-IPAA postoperative *de novo* CD incidence, prevalence and adverse events was performed. Publications regarding *de novo* CD in IPAA patients following proctocolectomy surgery for UC between January 1990 and December 2018 were searched. PubMed, MEDLINE, EMBASE (Excerpta Medica database), CINAHL, the Cochrane library, Web of Science, and Google^®^ were searched using the following terms: ulcerative colitis, total colectomy, proctocolectomy, restorative proctocolectomy, ileal pouch-anal anastomosis, *de novo* CD. Secondary and hand/manual searches of reference lists, other studies cross-indexed by authors, reviews, commentaries, books and meeting abstracts were also performed.

## 7. Results

Published data on *de novo* CD (pouch Crohn’s disease) developing after RPC with IPAA for UC and IC are presented in (Table 1). A total of 5042 IPAA patients were followed and ultimately 407 (8%) patients diagnosed with *de novo* CD are reported in the English literature to date (95% confidence interval (CI), 6.1–15.4%) after a median follow-up of 66 months (range: 3–236). All patients were diagnosed with clinically active pouch CD during follow-up surveillance after IPAA for UC or IC. Disease distinguishing traits significantly influenced pouch retention. The interval from pouch construction, increased incidence of nonblood diarrhea, disease fistulation, and location were used as prognostic indicators when *de novo* CD was diagnosed.

*De novo* CD diagnosis depended on either inflammation in the mucosa involving the small intestine proximal to the ileal pouch any time after IPAA surgery and/or when perianal complications developed more than 3 months after closure of a temporary diverting loop ileostomy. Time to diagnosis of *de novo* CD was widely defined as the time period from the closure of a diverting loop ileostomy. Alternatively, diagnoses associated to inflammatory or anatomic disorders of the ileal pouch afferent limb or efferent limb obstruction, pouch or anastomotic stricture, pouchitis, cuffitis, anal sphincter dysfunction, or *de novo* CD were evaluated using reported history, physical examination, pouch endoscopy and histologic interpretation. When there was a lack of findings and/or concern for alternative diagnoses (i.e., pouch leak, abscess or fistula), additional evaluation with imaging or examination under general anesthesia was reported as widely performed.

### Treatment of *de novo* CD:

**Figure 1:**
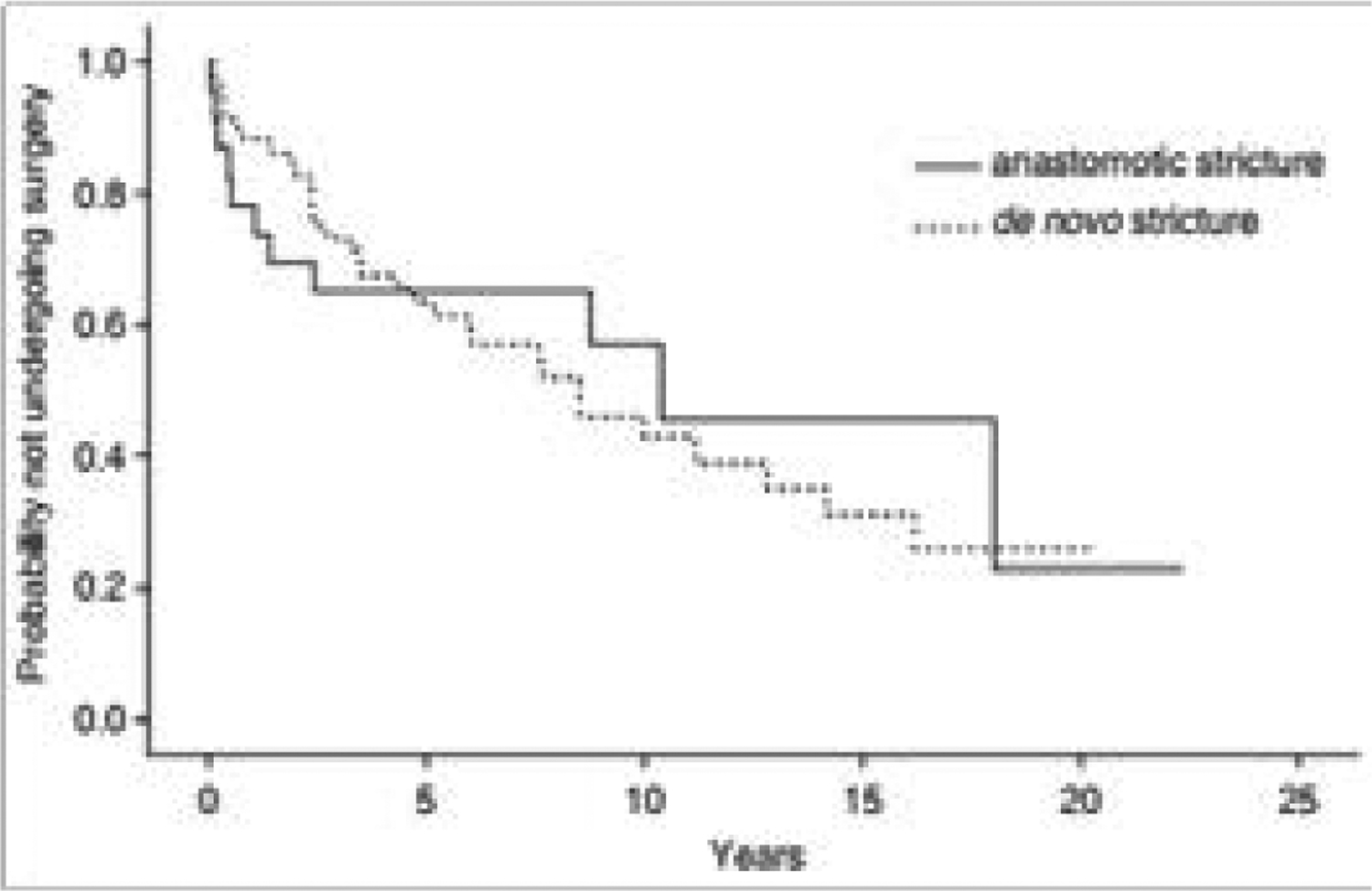
Kaplan-Meier estimated plot showing probability of surgery-free survival in relation to time after index dilatation in patients with anastomotic or de novo strictures (p = 0.86). This analysis is restricted to 83 patients having repeated dilatations only for strictures causing symptoms of bowel obstruction. Adapted with permission from “Endoscopic dilatation is an efficacious and safe treatment of intestinal strictures in Crohn’s disease”, by Gustavsson et al., Aliment Pharmacol Ther 2012;36:151–158 [24].

**Figure 2:**
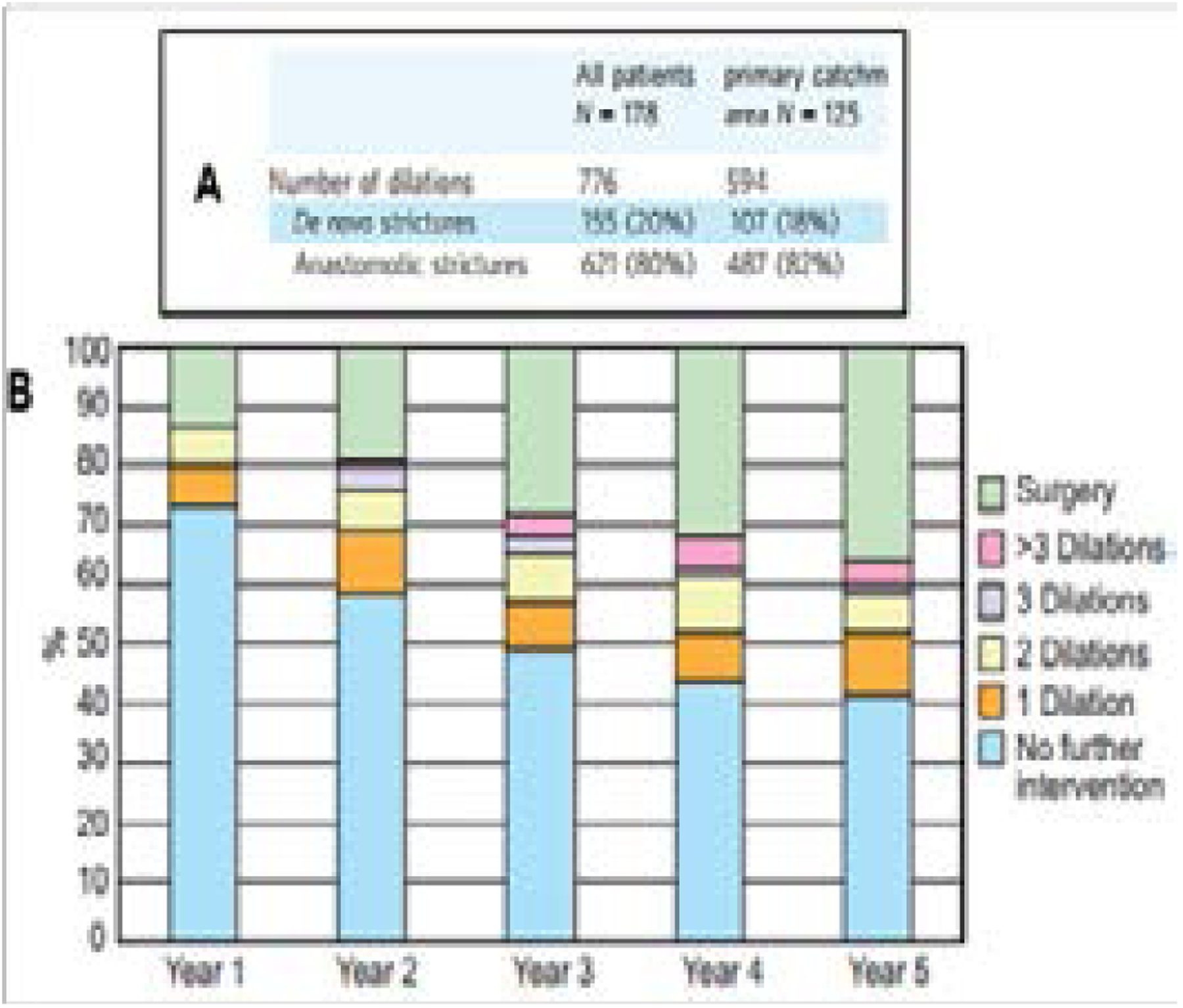
Kaplan-Meier estimated plot showing probability of surgery-free survival in relation to time after index dilatation in patients with anastomotic or F strictures (p = 0.86). This analysis is restricted to 83 patients having repeated dilatations only for strictures causing symptoms of bowel obstruction. Adapted with permission from “Endoscopic dilatation is an efficacious and safe treatment of intestinal strictures in Crohn’s disease”, by Gustavsson et al., Aliment Pharmacol Ther 2012;36:151–158 [24].

A firm diagnosis *de novo* CD often difficult to distinguish from that of chronic antibiotic resistant pouchitis or technical complications related to pouch surgery. Making an accurate diagnosis of *de novo* CD *vs*. other postoperative complications is important for, both, decisions regarding medical management and pouch excision *vs*. reconstruction. Most patients diagnosed with *de novo* CD are not considered for pouch reconstructive surgery [24]. There is very limited data available on the medical management of *de novo* CD and the quality of the studies are oftentimes execrable. Successful management is facilitated by co-operation within a multidisciplinary team of gastroenterologists and colorectal surgeons; and most importantly, closely involving the patient in therapeutic decisions with a person-centered approach and health coaching. Awareness of symptoms leads to timely consultation, diagnosis, treatment and restoration of intestinal continuity. The adapted representative data from Scandinavian [24], on cumulative proportions of patients undergoing surgery and time to surgery is shown in (figure 1). Surgical endoscopy interventions are depicted in (Figure 2A and B). Endoscopic balloon dilatation is an efficacious and safe alternative to surgical resection of a strictured *de novo* CD. About 52% of patients at 5-year follow-up require either no additional dilatation or only one additional dilation, where-as 36% required surgical resection.

### 7.1. Limitations:

According to the articles reviewed, the retrospective nature of these studies and Referral bias were limitations.

## 8. Discussion

**Figure 3:**
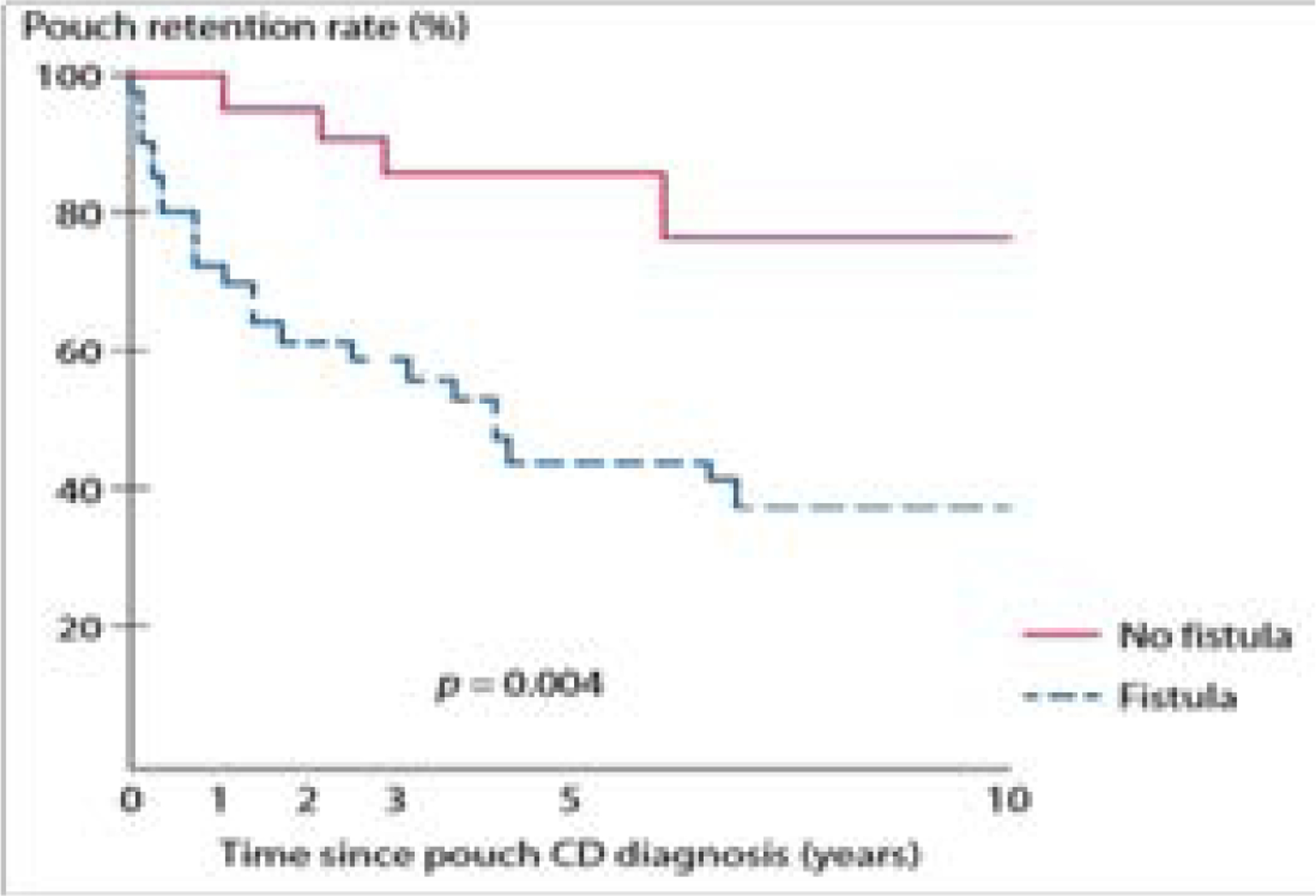
Kaplan-Meier estimated pouch survival in patients developing fistulas vs. without fistulas at the time of diagnosis of de novo Crohn’s disease. Adapted with permission from “Do clinical characteristics of de novo pouch Crohn’s disease after restorative proctocolectomy affect ileal pouch retention?” by Gu et al., Dis Colon Rectum 2014;57:76–82 [25].

**Figure 4:**
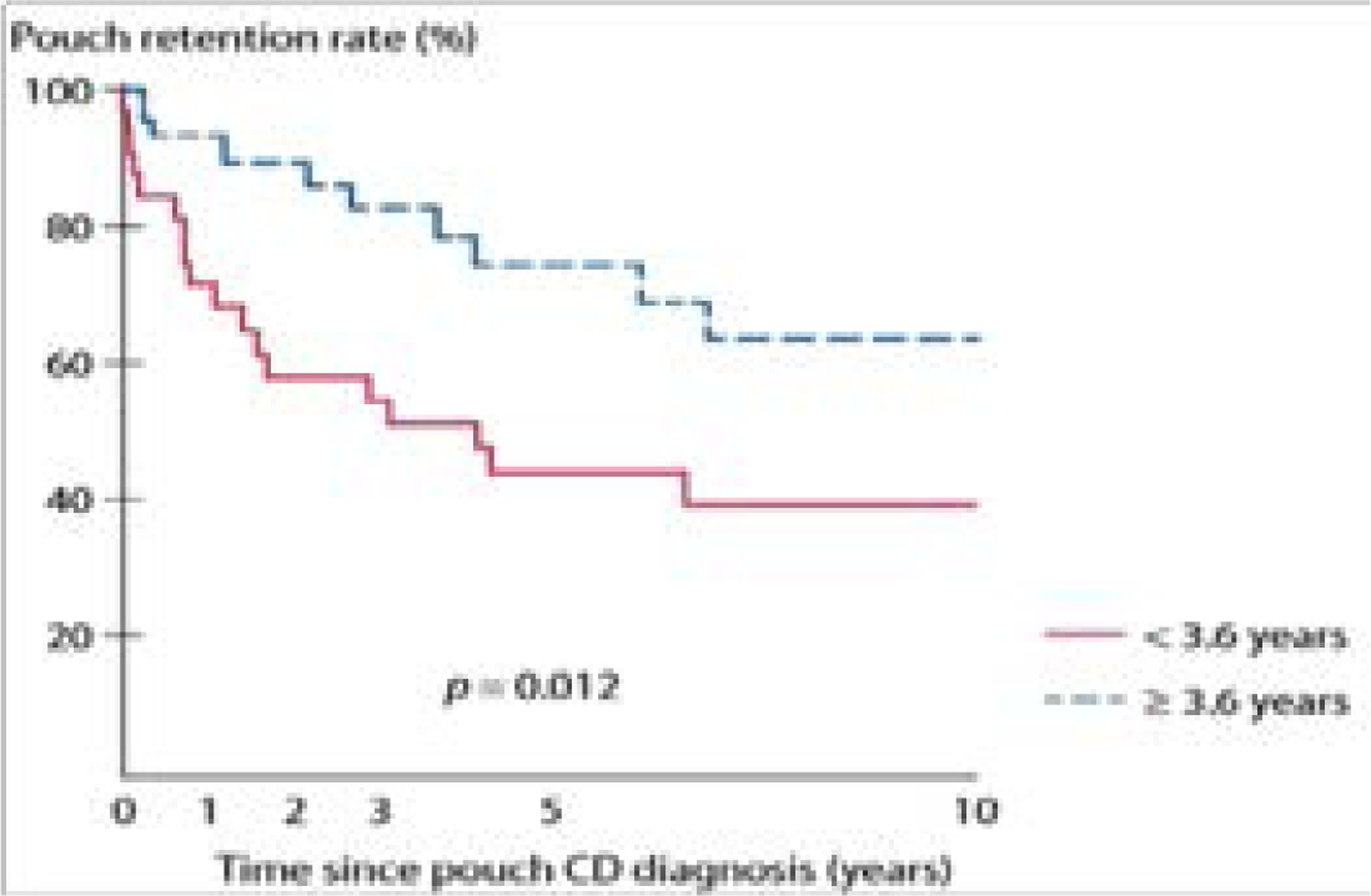
Kaplan-Meier estimated pouch survival in patients with different time interval from IPAA to de novo Crohn’s disease. Adapted with permission from “Do clinical characteristics of de novo pouch Crohn’s disease after restorative proctocolectomy affect ileal pouch retention? by Gu et al., Dis Colon Rectum 2014;57:76–82 [25].

Apprehension of the true incidence of the authentic *de novo* CD and standardizing the diagnostic criteria for this condition remain critical goals in improving the care of patients undergoing IPAA for the management of IBD. The incidence and prevalence of *de novo* CD after RPC with IPAA are addressed in many studies [8,14,15,25–27]. Out of the 13 projects herewith included, 12 studies included patients with UC and/or IC following IPAA and longitudinal analysis of patients who ultimately developed *de novo* CD [10,17,28–36]. A typical representative presentational result of *de novo* CD after RPC with IPAA is depicted by the Kaplan Meier in (figure 3) [25]. The estimated 10-year pouch retention rate in patients with a fistula at the time of *de novo* CD diagnosis was 38%; significantly lower than those without fistulae (44% vs. 76%, p = 0.004). Further, (figure 4) is representative data of early vs. late diagnosis of *de novo* CD [25]. The 10-year pouch retention rate significantly improved in patients with a late diagnosis of *de novo* CD (63% vs. 39%, p = 0.012). *De novo* CD leads to increased morbidity [27] and higher incidence of pouch failure [8,14,15,26]. Long-term observational studies of prospectively followed IPAA patients in a cohort from Cedar-Sinai Medical Center in Los Angeles, California [17], have found a higher rate of such pouch complications and pouch excision. Murrell at al., reported similar risk of *de novo* CD after IPAA after a median follow-up of 26 months [37]. Because *de novo* CD may develop over a longer follow-up period, the group evaluated the previously published IPAA cohort after the initial analysis (at 26 months) and showed an increase in new *de novo* CD by 19 per-cent during a median follow-up period of 69 months (range: 3–236) [17].

The studies highlighted the importance of distinguishing preoperative IBD (UV *vs*. CC) from postoperative IC patients when the diagnostic classification for these two diseases is inconclusive [1,37]. Based on The Working Party of the Montreal World Congress of Gastroenterology classification [38], maintained the preoperative and postoperative diagnostic classification [17], thereby overcoming several limitations of other studies that only used the postoperative diagnosis [8,11,12,39–44] of IC or UC when comparing outcomes after IPAA.

### 8.1. Is *de novo* CD after IPAA for UC a misdiagnosis of Crohn’s colitis?

Admittedly unknown, there are 3 possible causes that attempts to explain the development of *de novo* CD after IPAA for UC and IC: (i) authentic CD that was not evident prior to colectomy surgery, (ii) aberrated entity due to novel immunopathogenesis ingredient factors of the ileal pouch (antigen-antibody reaction against mucosal resistance), and (iii) a natural “transformation” of UC to *de novo* CD [9,45,46]. This last explains the “myth “of *de novo* CD; which lies on the observation that subsets of surgically untreated patients who are suffering from UC after initial definitive diagnosis work-up later develop features of CD allowing to change in original diagnosis [45]. These patients are thought to be “misdiagnosed^”^ [1]. This statement may not be wholly true given the fact that there is a possibility that these patients with authentic UC are “transformed”. Transformation occur because of an altered microbiome ecology in the ileal pouch [47], and immune environment [48], and subsequently the patient actually “converts”. Since there is evidenced overlap between clinical characteristics, epidemiological features, and disease associated genes/ genetic loci stratified by pathogenetic pathways and by disease location [49–51], it is possible that the authentic UC adhere to *de novo* CD within a given individual.

Pathway analyses highlighted differential gene expression with an up-regulation of Innate Immune Pathways (IIP) in CC and an up-regulation of Adaptive Immune Pathways (AIP) in UC [52].

In a recent report, PC specific peptide “Human alpha-defensin 5 (*DEFA5*)” differentiates the predominant subtypes of colonic IBD (CC vs. UC) [1]. *DEFA5* accurately identifies CC or UC phenotype among IC patients with a positive predictive value of 96 percent [1]. Furthermore, *DEFA5* can be used in selecting certain CC and UC patients that may have positive or negative outcomes after IPAA [1]. The distinction between UC and CC among patients with IC is of utmost importance when determining a patient’s candidacy for RPC and IPAA surgery [2,53,54].

### 8.2. Designating *de novo* Crohn’s disease at initial endoscopy biopsy

The overarching scientific premise is that UC patients likely to have positive or poor outcome, especially those that may transform/convert and adhere to *de novo* CD after IPAA based on Paneth cells (PCs) can be categorized using *DEFA5* expression during the first colonic mucosal endoscopy biopsy [55]. The significance is that we can anticipate patients with potential poorer outcomes from IPAA for *de novo* Crohn’s disease. Both observational and analytical data show that in colectomy specimens with an IC patient based on pathology criteria, the diagnosis can be resolved by quantitative IHC staining for *DEFA5* that predicts the actual, later diagnosis (CD *vs*. UC) as it becomes clinically apparent [1]. Admittedly, the diagnosis based on clinical criteria were correct for most IC patients, but the diagnoses were significantly delayed (7–14 years). Quantifiable *DEFA5* staining can clearly predict/ -classify the natural pathogenesis of IC to CC. The data in the retrospective cohort does specify classification in the initial (earliest) tissue specimen (pre-colectomy colonoscopy biopsy specimens that lead to the initial diagnosis of an IBD colitis). Whether *DEFA5* is merely an association or an actual driver of the *de novo* Crohn’s activity (or a marker for a mechanism of accelerated or refractory disease) remains to be elucidated. An important question is whether these observations are due primarily, or possibly secondary, to inflammation. The increase in the colonic *DEFA5* expression in CC patients correlated with the fecal calprotectin level (r = 0.481, p = 0.02) but not with the local histological inflammatory scores, suggesting that the general level of intestinal inflammation might trigger colonic Paneth cell metaplasia which is an additional protective mechanism in colonic inflammation [56,57]. It is noteworthy that clinical phenotype is the only clinical parameter that seems to be stable over time and does not change with disease progression (such as stenosis or fistulation) [58].

The etiopathogenesis of IBD is multifactorial and interplayed (e.g. immunological, genetic and environmental factors etc.) [59]. The action of dysregulation of PCs in IBD has very important mechanistic implications because it leads to dysregulation of the secretion of *DEFA5* and other antimicrobial peptides which are vital in the checks and balances regulation of gut microbes and homeostasis. Understanding specific signaling pathways of the colonic immunological system is therefore important. Perminow et al. has described “defective Paneth Cell-mediated host defense in ileal CD” [57]. They also demonstrate disparity of *DEFA5* between small vs. large bowel CD and correlated this with fecal calprotectin levels. In the study, they describe a specific decrease of *DEFA5* levels and TCF-4 mRNA expression in ileal CD. This decrease was seen to be independent of inflammation, whereas inflammation seemed to induce PC metaplasia in the colon [57]. The data extended the hypothesis of the role of antimicrobial host defense in CD patients. Similar observations were reported. [60]. These studies report the mechanisms of intestine luminal immune response, with nothing about the diagnostic accuracy and/or predictive subsequent *de novo* CD in colonic biopsies prior to surgery with RPC and IPAA. We have demonstrated the detection of *DEFA5* in patient tissues and sera as a specific, and more reliable, diagnostic tool to distinguish CC from UC. With this tool, we have validated that misdiagnoses of IBD as IC cases will be circumvented and appropriate care can be provided to the hitherto IC classified patients [1]. We also anticipate that data from this study will provide a strong basis to explore the possibility of *DEFA5* and/or disease specific cytokines as a potential therapeutic target for CC. Florian Kuhn et al. describes surgical principals in the treatment of UC [61]. We published this information 10 years earlier and agree [2]. However, there is no information on the diagnosis accuracy or predictive subsequent *de novo* CD. We have demonstrated, in real patients, the diagnostic accuracy and subsequent predictive *de novo* CD prior to surgery with RPC and IPAA [1].

## 9. Article Highlights

### 9.1. Background:

The *de novo* CD is one of the most common long-term complication of IPAA and a leading cause of pouch failure and pouch excision or permanent diversion. So, it is desirable to find preoperative clinical characteristics that can predict eventual outcome of patients with UC prior to IPAA who would develop *de novo* CD. This will allow patient personalized surveillances.

### 9.2. Motivation:

Aimed at identifying a unique biomarker that efficiently distinguish CC from UC among IC patient cohorts (at first endoscopy biopsy). The use of *DEFA5* like a marker that can select certain UC (and CC) patients who may have positive or negative outcomes after IPAA, would be critically important for choosing the correct surgical strategy, avoiding to subject patients with CC, to a harmful surgical treatment.

### 9.3. Methods:

We performed literature search based on PRISMA-P, between 1990 and 2018.

### 9.4. Results:

*DEFA5* bioassay is a predictive and diagnostic test specific for *de novo* CD and CC. Patients who develop *de novo* CD are preoperatively thought to have a “*misdiagnosed*” CC. This claim remains skeptical given the fact that there is a possibility that these patients had a truly authentic UC but are “*transformed*” and convert to adhere to *de novo* CD. Natural transformation occurs because of an altered microbiome ecology in the ileal pouch, and immune environment, and subsequently the patient actually “*converts*”.

### 9.5. Conclusions:

*DEFA5* has shown the ability to delineate CC and UC phenotype and circumvents indeterminate Colitis (IC) into accurate authentic UC or CC. *DEFA5* may be used to predict *de novo* CD prior to and can select CC patients who are likely to have positive OUTCOME after IPAA surgery. *DEFA5*, is detectable in circulating human blood.

### 9.6. Perspective:

*DEFA5* bioassays, if successfully developed may provide an important translational accessory for improved IBD diagnosis, initial assessment prior to surgical intervention and more importantly, prescription of disease subtype appropriate treatment options. Future endeavors will AIM to determine the molecular basis of stem cell differentiation in the crypt of CC patients, and if disease severity depend on the levels of secreted *DEFA5* and/or specific pro-inflammatory cytokines.

## Figures and Tables

**Table 1:** Depicts the thirteen full-text publications included in Overview of de novo Crohn’s disease Incidence. Full publications included in Overview of de novo Crohn’s disease Incidence

Study Authors	Publication Year	Study Period	Number of IPAA Patients in Studies	Number of De novo CD	*De novo* CD Incidence in %
Goldstein et al. [27]	1997	1981–1995	74	8	10.8
Peyregne et al. [28]	2000	1985–1997	43	4	9.3
Rossi et al. [29]	2002	1989–2000	68	4	5.9
Melton et al. [30]	2010	1983–2007	2814	87	3.1
Haveran et al. [31]	2011	1990–2009	382	32	8.3
Coukos et al. [32]	2012	2006–2010	142	21	14.8
Tyler et al. [33]	2012	N/A-2007	399	50	12.5
Ahmed et al. [8]	2016	1992–2014	199	42	21
Zaghiyan et al. [15]	2016	1997–2007	237	40	16.9
Diederen et al. [34]	2017	2000–2015	303	14	4.6
Lightner et al. [16]	2017	1982–2016	Unreported	35	N/A
Yanai et al. [35]	2017	1981–2013	253	54	21.3
Shamah et al. [26]	2018	1960–2015	128	16	12.5
**Total**	**1997–2018**	**58 Years**	**5042**	**407**	**3.1**–**21.3% (mean 11.75)**

## 4. Core tip:

Due to lack of specificity, applicable robust preoperative risk factor(s) predicting *de novo* Crohn’s Disease (CD) have to date not been identified. Appreciating the incidence of *de novo* CD and bring into conformity with standard diagnostic criteria remains critical challenge and goals in the surgical care of patients with ulcerative colitis (UC) and/or Indeterminate Colitis (IC) indicated Restorative Proctocolectomy (RPC) and Ileal Pouch-Anal Anastomosis (IPAA). Approximately 15% of patients diagnosed with UC and another 15% of IC patients who are surgically treated with RPC and IPAA resolve with an ultimate painstaking devastating complication, the *de novo* CD, which often cause significant morbidity and pouch failure. Several of these patients may not respond favorably to conversional conservative medical treatment, ultimately requiring surgical revision or permanent fecal diversion. These patients are preoperatively thought to have a “*misdiagnosed*” Crohn’s Colitis (CC). This claim remains skeptical given the fact that there is a possibility that these patients had a truly authentic UC but are “*transformed*” and convert to adhere to *de novo* CD. Natural transformation occurs because of an altered microbiome ecology in the ileal pouch, and immune environment, and subsequently the patient actually “*converts*”. In certain cases, authentic CD are not evident prior to proctocolectomy surgery and/or gradually manifest/acquire an aberrated novel immunopathogenesis ingredient factors of the ileal pouch (antigen-antibody reaction against the mucosal resistance) of individual patients. Crypt Paneth cell (PC) secreted antimicrobial peptide, Human alpha-defensin 5 (*DEFA5* or HD) has shown the ability to delineate UC from CC and that circumvents IC into actual diagnosis of UC or CC at initial clinic visit endoscopy biopsy procedures, a realizable potential that might subdue diagnostic delay in the IBD Clinic. Furthermore, *DEFA5* denotes UC patients prior to colectomy likely to transform and convert to *de novo* CD after IPAA surgery. We also propose that *DEFA5* may permit selection of certain subtype of CC patients who are likely to have a positive outcome following IPAA surgery. Promising data are underway.

## Abbreviations:

IBD: Inflammatory Bowel Disease
UC: Ulcerative colitis
CD: Crohn’s disease
CC: Crohn’s colitis
IC: Indeterminate colitis
MOOSE: meta-analyses of observational studies
RPR: Restorative proctocolectomy
IPAA: Ileal pouch-anal anastomosis
PRISMA-P: Preferred reporting items for systematic review and meta-analysis Protocols
MEDLINE: Medical Literature Analysis and retrieval system online
EMBASE: Excerpta Medica database
CINAHL: Cumulative index of Nursing and Allied Health Literature
SCFAs: Short-chain fatty acids
NOD2: Nucleotide-binding oligomerization domain-containing protein 2
TNF-α: Tumor necrosis factor-alpha
DEFA5: Human Alpha-defensin 5 (Paneth cell specific)
PC: Paneth cell
